# Trichothecene Genotypes of *Fusarium graminearum* Populations Isolated from Winter Wheat Crops in Serbia

**DOI:** 10.3390/toxins10110460

**Published:** 2018-11-08

**Authors:** Vesna Krnjaja, Slavica Stanković, Ana Obradović, Tanja Petrović, Violeta Mandić, Zorica Bijelić, Manja Božić

**Affiliations:** 1 Institute for Animal Husbandry, Autoput 16, 11080 Belgrade-Zemun, Serbia; violeta_randjelovic@yahoo.com (V.M.); zonesh@gmail.com (Z.B.); 2 Maize Research Institute “Zemun Polje”, Slobodana Bajića 1, 11185 Belgrade, Serbia; sstojkov@mrizp.rs (S.S.); aobradovic@mrizp.rs (A.O.); 3 Institute of Food Technology and Biochemistry, Faculty of Agriculture, University of Belgrade, Nemanjina 16, 11080 Belgrade-Zemun, Serbia; tpetrovic@agrif.bg.ac.rs; 4 Faculty of Biology, University of Belgrade, Studentski trg 16, 11000 Belgrade, Serbia; manja.z.bozic@gmail.com

**Keywords:** winter wheat, Fusarium head blight, *Fusarium graminearum*, trichothecene genotypes

## Abstract

*Fusarium graminearum* as the main causal agent of Fusarium head blight (FHB) and its ability to produce trichothecenes was investigated by molecular techniques. A total of 37 strains isolated from the wheat, harvested in Serbia in 2005, 2008 and 2015, and previously designated by morphological observation as *F. graminearum*, were used for trichothecene genotypes characterization. The strains were identified using the species-specific primer set FG16R/FG16F while genotypic characterization was done using specific TRI13 and TRI3 sequences of the trichothecene gene clusters. The PCR assays identified all strains as species of *F. graminearum sensu stricto* with the DON/15-ADON genotype. The quantification of the mycotoxin (DON) was performed using the biochemical assay. The high levels of DON (>20,000 µg kg^−1^) were recorded in all of the strains from 2005, four strains from 2008 and two strains from 2015. Weather data of the investigated seasons, showed that the optimal temperature, frequent rains and high relative humidity (RH) was very favourable for the development of *F. graminearum*, affecting the DON biosynthesis.

## 1. Introduction

Wheat (*Triticum aestivum* L.) is cultivated in Serbia on approximately 600,000 ha with the average yield of 4.8 t ha^−1^ in 2016 according to data of Serbian Statistical Yearbook. As a staple food, it is primarily used in the production of flour, semolina, bread, baked food, cakes, pasta etc. In addition, it can be used as concentrated feed in animal husbandry. This crop is a rich source of carbohydrate, proteins, polyunsaturated fats, α-tocopherol (vitamin E), vitamins of group B, dietary fiber, minerals, phytochemicals and antioxidants in the human diet.

Fusarium head blight (FHB) or head scab is the main disease of wheat (*Triticum aestivum* L.) worldwide [1]. This disease is caused by the complex of species of the genus *Fusarium*, wherein the incidence of certain species depends on a geographic region. *Fusarium graminearum* Schwabe (*Gibberella zeae* (Schwein.) Petch) and *Fusarium culmorum* (Wm. G. Smith) Sacc. are primary causal organisms of FHB in Europe. *F. graminearum* and *F. culmorum* are the predominant *Fusarium* spp. in Europe with the former commonly isolated in southern (warmer) and the latter in northern (colder) European domain [2,3]. The occurrence of FHB by *F. graminearum* in Serbia is a consequence of favourable agro-ecological conditions and moderate-continental climate [4].

In addition to extensive yield and quality losses of wheat, a great concern is the potential of *F. graminearum* to produce mycotoxins, in particular type B trichothecenes such as deoxynivalenol (DON) and nivalenol (NIV) along with their acetylated derivatives [5]. More acetylated derivatives of DON, 3-acetyldeoxynivalenol (3-ADON) and 15-acetyldeoxynivalenol (15-ADON) than the acetylated derivatives of NIV, 4-acetylnivalenol (4-ANIV or fusarenon X) were detected in the grains infected with *F. graminearum*. The incidence of these genotypes/chemotypes varies by geographic region. Thus, the occurrence of both genotypes (DON and NIV) is recorded in certain countries of Africa, Asia and Europe, while DON genotype occurs only in North America [6]. The Both genotypes of these mycotoxins have been identified within some strains of *F. graminearum*. A correlation between a geographical origin and occurrence of particular genotypes was established in *F. graminearum* strains that produced 3-ADON or 15-ADON. Accordingly, strains from China commonly produce 3-ADON, while strains derived from Mexico, North America and Europe mainly produce 15-ADON [7].

Trichothecenes may cause health disorders in humans and animals, especially when ingested in high amounts. Exposure to trichothecenes can cause feed refusal, intestinal disorders, vomiting, skin dermatitis, hemorrhagic lesions and immunological problems [8]. In addition, contamination of wheat grains with DON and NIV pose negative effects on the economy in food and livestock production [9].

Parry et al. [2] and Hooker et al. [10] reported that weather conditions in the period from pollination to maturation had strong impacts on grain infection with FHB and production of trichothecene during harvest. Therefore, it is of great importance to develop forecasting disease models based on the monitoring of weather conditions in order to disseminate information to farmers about the preventive measures against FHB disease and potential infection of grains with mycotoxins [11]. The risk assessment of the effects of disease is best expressed over the parameters for yield and grain quality. The levels of mycotoxins in the grains as well the isolation and molecular identification of *Fusarium* species as the main causative agents are also useful parameters of the severity of FHB infection [12]. One of the methods of developing the FHB monitoring model based on weather forecast is through a risk assessment over a period of several years. The probability of occurrence of harmful fungi and their potential to produce mycotoxins, can be estimated based on a given locations and weather conditions, which may contribute to both, the risk assessment of wheat contamination and the impact on food safety.

Although the mechanisms of tolerance to FHB and DON accumulation in wheat grain are complex and not sufficiently illuminated [13], FHB control encompasses the use of tolerant wheat varieties [14] as well as the application of chemical (triazole-based fungicides) [15] and biological protection measures [16].

ELISA and LC/MS are the conventional analytical methods used for determination of trichothecene concentrations of *F. graminearum* strains, while advanced PCR methods are used for amplification of trichothecene biosynthesis genes of *F. graminearum* strains that produce particular genotypes [17,18,19]. The biosynthesis of *Fusarium* trichothecenes is controlled by the 12-gene core *TRI* cluster. *TRI13* and *TRI7* are the two most important genes for determination of the production of the DON or NIV genotype. The conversion of DON to NIV in *Fusarium* is controlled by the *TRI13* gene, while the *TRI7* gene is responsible for acetylation of NIV to produce 4-ANIV [20]. PCR methods are used to determine the differences among the functionality and non-functionality of *TRI13*. Therefore, the *TRI13* gene is one of the most commonly used genetic markers for the identification of 3-ADON, 15-ADON and NIV genotypes in PCR assays [21].

Regular analyses of genetic and toxigenic properties of main causal agents of FHB are important for the estimation of potential risks of occurrence of mycotoxins in wheat grains in a certain geographical region. Therefore, the aim of these studies was to apply the PCR assays in order to determine genetic diversity and trichothecene genotyping of *F. graminearum* (37 strains) isolated from the winter wheat grains, harvested in the vicinity of Belgrade, Serbia, during the three different growing seasons (2005, 2008, and 2015). In addition, the ability of 18 randomly picked strains to produce DON was also investigated, as well as the influence of climatic factors (temperature, rainfall and relative humidity) on genetic and toxigenic potential of *F. graminearum* populations.

## 2. Results

### 2.1. Climatic Conditions

According to the data supplied by the Republic Hydro-Meteorological Service of Serbia, higher temperatures and rainfalls were recorded in Belgrade area during the May, coinciding with the period of wheat anthesis where *Fusarium* species may cause infections of the spikes. The mean monthly temperatures and monthly rainfalls were 17.7, 19.3, and 19.1 °C and 47.4, 60.6, and 80.7 mm, for 2005, 2008, and 2015 respectively.

**Figure 1:**
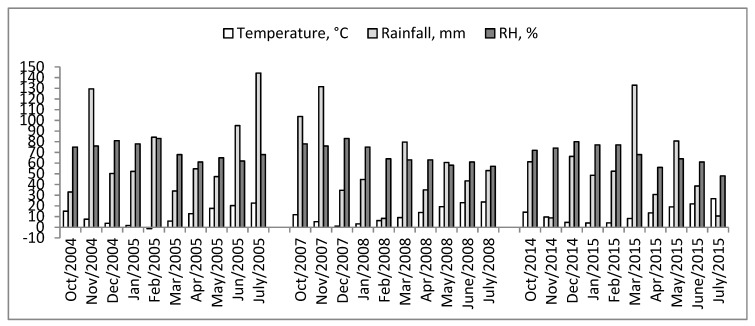
Mean monthly temperature, total monthly rainfall and mean relative humidity (RH) from October to July for seasons 2004–2005, 2007–2008 and 2014–2015 in the Belgrade area.

However, during the period of wheat anthesis in June, and maturation and ripening stage in July, rainfalls were significantly higher in 2005 than in 2008 and 2015. The monthly rainfalls for June and July were 95.1 and 144.3 mm, 43.3 and 53.0 mm and 38.6 and 10.6 mm, respectively, whereas the mean monthly temperatures were generally >20 °C and relative humidity (RH) >60% (Figure 1). Moreover, observing the wider environmental data from the start of the growing season from October to July, the total season rainfall and seasonal RH mean values were also higher in the growing seasons of 2004–2005 than in 2007–2008 and 2014–2015. The total season rainfall and mean RH were 724.5, 594, and 530.8 mm and 71.7%, 67.8%, and 67.7%, respectively.

### 2.2. Morphological and Molecular Identification of Species

**Figure 2:**
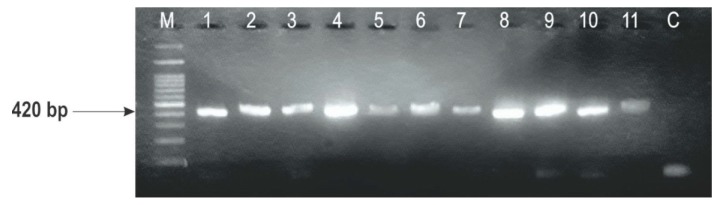
Amplification of PCR products with species-specific primers FG16F/FG16R for the identification of *Fusarium graminearum*; lane M, 100 bp DNA ladder and lane C, control.

Based on the appearance of colony and macroconidia, the fungal strains isolated from wheat grains have been identified as *Fusarium graminearum*. Among them, 37 strains were picked from fungal collection of the Institute for Animal Husbandry, Belgrade for molecular identification with PCR method. All of the tested strains yielding the product of amplification of 420 bp, confirming that they belong to the species of *F. graminearum sensu stricto* (s.s.) (lineage 7) (Figure 2).

### 2.3. Identification of F. graminearum Genotypes

**Figure 3:**
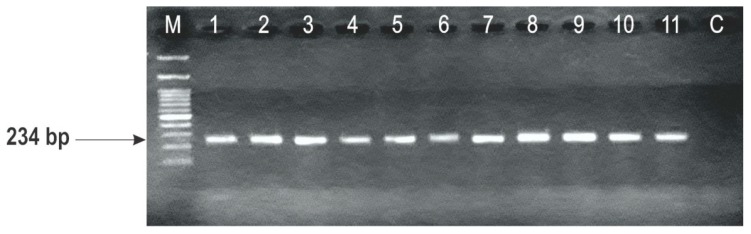
Amplification PCR products with primers TRI13F/TRI13R specific to deoxynivalenol (234 bp) and nivalenol (415 bp) genotypes; lane M, 100 bp DNA ladder and lane C, control.

The possibilities of *F. graminearum* strains to have DON and NIV genotypes were performed by another PCR assay with the primer pair TRI13F/TRI13R. The tested primer sets enabled identification of DON producers in all of the tested *F. graminearum* strains, with the fragment of amplification of 234 bp (Figure 3).

**Figure 4:**
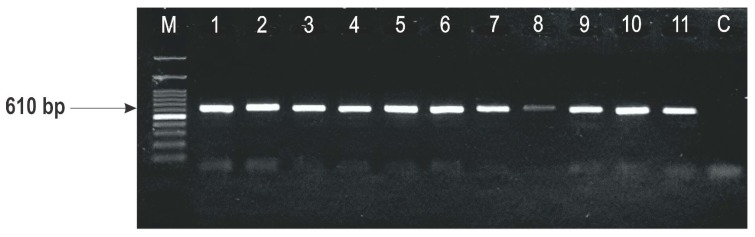
Amplification PCR products with primers TRI3F/TRI3R specific for 4-acetylnivalenol (840 bp), 15-acetyldeoxynivalenol (610 bp), and 3-acetyldeoxynivalenol (243 bp) genotypes; lane M, 100 bp DNA ladder and lane C, control.

Results of the multiplex PCR assays with primers specific for identifying acetylated derivatives revealed the presence of only 15-ADON (Table 1), giving the PCR product of amplification of 610 bp (Figure 4).

### 2.4. Deoxynivalenol Production

The ability of tested strains to produce DON was evaluated in 18 randomly picked strains (six strains per year), under in vitro conditions, and results showed that selected strains were DON positive, with highest toxigenic potential of >25,000 µg kg^−1^ recorded in the three strains of 2005, followed by two strains isolated in 2008 and one strain isolated in 2015. The production of DON was not detected in the control sample, where the DON level was below the detection limit of 40 µg kg^−1^ (Table 2).

## 3. Discussion

In this study, the presence and frequency of trichothecene genotypes and their acetylated derivatives among *F. graminearum* populations were established by different PCR analyses. In addition, the results on the toxigenic potential of 18 randomly picked *F. graminearum* strains to produce DON have been presented.

The first experimental identification of the strains was previously carried out using microscopic and morphological investigation (colony and conidial morphology). Based on these observations, they have already been designated as *F. graminearum*. In order to perform reliable identification on the molecular level, the primer set FG16F/FG16R was used in the current work and the product of amplification of 420 bp was obtained, confirming that all the strains belong to *F. graminearum sensu stricto* (*F. graminearum* s.s.). Within the *Fusarium* spp., *F. graminearum* has already been isolated and morphologically identified, under agro-ecological conditions of Serbia, as the major species associated with FHB in winter wheat varieties [4,22]. Additionally, other *Fusarium* species that are less pathogenic or opportunistic, such as *F. oxysporum*, *F. poae*, *F. proliferatum*, *F. semitectum*, *F. sporotrichioides*, *F. subglutinans*, and *F. verticillioides* have been determined, but with a lower incidence [23].

This study demonstrated that all the strains were DON genotype producers (234 bp), testing with the primer set developed for the *TRI13* gene, whereas with another primer sets for the *TRI3* gene, the 15-ADON genotype was clarified. In previous molecular studies of the presence of trichothecene genotypes in *F. graminearum* strains isolated from the wheat, maize and barley in Serbia, Obradović et al. [24] have also reported the occurrence of DON and its 15-ADON derivative. This survey represents the second report of the trichothecene genotyping of *F. graminearum* in Serbia, but included three different varieties of wheat and strains isolated during three different growing seasons, covering a relatively long distance period (2005, 2008 and 2015). Compering these results, based on the detected genotypes, there was no supposed genotype diversity between maize, barley and wheat, indicating that genotype diversity within *F. graminearum* is not mainly associated with the host. This was in line with the observation of Boutigny et al. [25] for the genotype diversity among the maize and wheat as host cultures in France.

The current study confirmed that FHB in Serbia was associated only with the 15-ADON genotype, while 3-ADON and NIV were not recorded. NIV producer has not been found in the current study, but has been found in one strain of *F. graminearum* isolated from the wheat crops in Romania and Turkey [26,27]. Additionally, Szécsi et al. [28] have reported that *F. graminearum* strains originating from Hungarian cereal and maize-growing regions mainly belong to the DON genotype, but that the majority of them had both 3-ADON and 15-ADON and only at one detected DON/NIV genotype. Interestingly, these authors have presumed that the variability between genotypes was independent of geographical and environmental conditions in Hungary, and supposed that these were caused by specific regional contamination of the wheat grain. The high proportion of DON genotypes within the *F. graminearum* s.s. has also been reported in the survey of Bilska et al. [29] who found 15-ADON genotypes in more than 80% of wheat samples from Poland. In the region of northern Italy, all three fungal genotypes among *F. graminearum* strains were isolated from durum wheat, with the 15-ADON as the most frequent (87.2%), followed by the 3-ADON (8.1%) and the NIV genotype (2.7%) [30]. Similar results were obtained by Covarelli et al. [8] in central Italy, and Talas et al. in Germany [31]. Likewise, in certain years, in the Netherlands, UK, Belgium, and Poland the increased proportion of NIV producers (>15%) were identified in the wheat [25]. A high occurrence of NIV should be of concern, as NIV is potentially more toxic to humans and animals compared to DON. Interestingly, unlike the DON producers, NIV have occurred regardless of climatic conditions, pointing that it could often be an under-estimated risk to be further analyzed [8].

In addition to European countries, the dominance of the DON/15-ADON genotype of *F. graminearum* s.s. isolated from the wheat crops has also been confirmed in southern Brazil [32], Argentina [33], South Africa [34], China [20,35], and in Taiwan [19]. In some countries, there are certain differences in the regional occurrence and distribution of DON genotypes. The predominance of 15-ADON producers of *F. graminearum* s.s. have been reported in Midwestern USA [36] unlike southern Louisiana where a high level of NIV genotypes has been found [37]. Regional differences between DON genotypes have also been observed in Japan, Russia and Iran, and the 15-ADON genotype of *F. graminearum* populations was dominant in the northern part of Japan, southern part of Russia and north-west of Iran [38]. Additionally, in western Canada, 3-ADON *F. graminearum* producers were replaced by 15-ADON producers [39]. Interestingly, Beyer et al. [40] supposed that changing from NIV-producing *F. culmorum* strains to 15-ADON-producing *F. graminearum* in Luxembourg were associated with better competitiveness of the latter strain to humid climate conditions.

The main reason for the differences in the distribution of DON and NIV genotypes in *F. graminearum* population is generally unknown but it could be possible that these might be a consequence of variations in climatic conditions, soil type, host genotype, applied cropping practices, such as soil tillage, fertilization, irrigation, fungicides, crop rotation and also condition during grain storage.

Quantitative analysis revealed a high toxigenic potential of identified strains to produce DON, with an amount that exceeded the 20,000 µg kg^−1^, in all six, four and two *F. graminearum* strains originated from 2005, 2008, and 2015, respectively. In previous research of Stanković et al. [41] significant levels of DON were also quantified in *F. graminearum* strains, isolated and morphologically identified, from the wheat grains collected from various regions in Serbia. Their results indicate that these high levels ranging from 160 to 45,260 µg kg^−1^ were regionally dependent. Under similar study conditions, conducted by Obradović et al. [24], the high DON levels from 23,800 to 88,700 µg kg^−1^, have also been recorded in wheat, confirming this assumption. Likewise, significant levels of DON were documented in Italian durum and soft wheat, where the higher average DON contamination was found in durum wheat than in the soft wheat, whereas NIV was evenly detected at significant concentrations in both wheat species [8]. Interestingly, different degrees of mycotoxins reduction could be accomplished during processing of food including sorting, cleaning, milling, baking etc. [42]. For example, DON is particularly concentrated in the bran coat which is eliminated in the production of flour and semolina, contributing to its lower value in the final food products [43].

In this survey, the climatic factors (temperature, rainfall and RH) did not affect diversity of trichothecene genotypes of *F. graminearum*, since the DON/15-ADON was the only genotype isolated. The temperatures during anthesis stage, in May (>17 °C), and June (>20 °C), contributed to development of *F. graminearum* populations in the grains of infected wheat, whereas high seasonal total rainfalls and seasonal mean RH were favorable for the production of DON and its 15-ADON derivatives. This is consistent with the results of Prodi et al. [44], who also documented the dominance of 15-ADON genotypes of *F. graminearum* in humid areas of northern Italy. However, Boutigny et al. [25] have found regional differences in the distribution of 15-ADON and NIV genotypes, with higher incidence of NIV-*F. graminearum* strains in the warmest area (South France). Likewise, Zhang et al. [35] recorded the increase presence of 15-ADON-*F. graminearum* genotypes in the colder wheat growing areas of China with the annual average temperatures of 15 °C or lower. In addition to the dominance of the DON/15-ADON genotype, high total rainfalls during anthesis and maturation, in June (95.1 mm) and July (144.3 mm) recorded in 2005, provoked the strains of *F. graminearum* isolated in that year to produce a higher level of DON than those of 2008 and 2015, which correlated well with the findings of Audenaert et al. [45] in Belgium. These authors found a positive correlation between the accumulation of DON in winter wheat and high amounts of rainfall (210 mm) in June of 2007, during and after the stage of anthesis. Similarly, in Argentina, Alvarez et al. [46] found different values of toxigenic potential for the production of DON, depending on the year of investigation, whereas Covarelli et al. [8] confirmed that climatic factors mainly affected the composition of fungal toxigenic species, and the level of mycotoxin contaminations in soft and durum wheat grains in central Italy.

## 4. Conclusions

The 37 strains isolated from three wheat varieties, and previously designated as *Fusarium graminearum*, were confirmed to belong to the species *F. graminearum sensu stricto* (*F. graminearum* s.s.), using precise molecular assay. Multiplex PCR assay revealed the presence of only DON/15-ADON genotypes in all of the tested strains, with the product of amplification of 610 bp. The concentrations of DON were quantified by ELISA method, were high level (>20,000 µg kg^−1^), and was recorded in 12 of 18 randomly picked strains. Considering the optimal temperature for the development of *F. graminearum* during the wheat anthesis (<17 °C), as well as another stimulated condition (frequent rain and high relative humidity), had a crucial impact on the dominance of the DON/15-ADON genotype as expected. Likewise, abundant rainfall in the tested location (Belgrade area) in June and July, especially in 2005, during and after anthesis, resulted in a high potential of all the *F. graminearum* strains, isolated from that year, to produce DON.

The results from this survey indicate that the combined approach might contribute to rapid and precise identification of *F. graminearum* and the presence of trichothecene genotypes, as well as DON producers in wheat crops. Thus, an integrated disease management strategy is necessary in order to minimize economic losses and mycotoxin contamination with negative impact on human and animal health. The knowledge about *F. graminearum* population and genotype diversity and distribution could be of great importance for development of forecasting disease strategies on a regional level. This would be useful tool in early detection of trends and potential hazard of trichothecene *Fusarium* species. Further investigation should focus on more strains from other regions of Serbia in order to obtain more comprehensive insight on the *F. graminearum* populations at a national level.

## 5. Materials and Methods

### 5.1. Fungal Strains

The samples of the local varieties of winter wheat (Pobeda, Simonida, NS40S) were taken from the production plots of the Institute for Animal Husbandry, Belgrade-Zemun, during the harvest in 2005, 2008 and 2015. According to the morphological (macroscopic and microscopic) observation, the strains were identified as *F. graminearum* and kept in the fungal collection. The 37 single-spore *F. graminearum* were used in this investigation to confirm their identification on molecular level by advanced PCR methods.

The data information about the origin of the strains and year of isolation are shown in Table 1.

All of the isolated single-spore *F. graminearum* strains (37) were taken for these investigations, and cultivated on potato dextrose agar (PDA, Biolife, Milano, Italy), in order to analyze their potential to produce trichothecene genotypes.

### 5.2. DNA Extraction

*F. graminearum* strains were grown on PDA (potato dextrose agar, Biolife, Milano, Italy) and incubated at 25 °C for 7 days (Memmert incubator, Schwabach, Germany). DNA was extracted from the fungal mycelium according to the manufacturer’s instructions (DNeasy Plant Mini Kit, Qiagen, Hilden, Germany). The extracted DNAs were stored at −20 °C, prior to the analysis.

### 5.3. Polymerase Chain Reaction

A total of 37 strains were identified using the primer pair FG16F/FG16R, which produced polymorphic products only with DNA of *F. graminearum*, but did not amplify with DNA of closely related *Fusarium* species or fungi of other genera [47]. In order to analyze the potential of tested strains to produce trichothecene genotypes, this was based on the production of DON and NIV using the primer pair TRI13F/TRI13R for the *TRI13* gene [48]. The multiplex PCR reaction was performed with the primer sets 3CON, 3NA, 3D15A, and 3D3A for the *TRI3* gene [49] for identifying acetylated derivatives (Table 3).

The amplification reactions were conducted using 1.1 µL of fungal DNA (10–20 ng) in a total volume of 20 µL containing, 2 µL of 10 × buffer PCR (100 mM tris-HCL, 15 mM MgCl_2_, 500 mM KCl, pH 8), 1.2 µL of MgCl_2_ (25 mM), 0.4 µL of dNTPs (10 mM), 0.1 µL of Taq DNA polymerase (5 U/µL) and 1 µL of each primer (20 mM). All the PCR reactions were performed in a thermocycler (Quantarus, UK). The conditions for the amplification of the *FG16* gene was as follows: Denaturation step at 94 °C for 30 s, 30 cycles of 95 °C (30 s)/57 °C (30 s)/2 °C (60 s), and a final extension at 72 °C for 5 min. For the amplifications of the *TRI13* gene and for the multiplex PCR reaction of *TRI3* genes, the amplification conditions were as follows: Denaturation step at 95 °C for 5 min, 40 cycles of 95 °C (30 s)/57 °C (30 s)/72 °C (1 min), and a final extension at 72 °C for 10 min.

The expected fragments of 400–500 bp for *FG16*, 234 or 415 bp for *TRI13* and 840, 610 and 243 bp for *TRI3* were separated on 1.5% agarose gel in 1 × TBE (Tris-borate-EDTA) buffer, and visualized under UV light on a transilluminator (UV-transilluminator, MD-20, 312 nm, Wealtec Corp., Sparks, NV, USA). DNA was stained with Midori Green (Nippon Genetics Europe, Dueren, Germany). The 100 bp DNA ladder (Nippon Genetics Europe, Dueren, Germany) was used as a molecular marker.

### 5.4. Deoxynivalenol Production

The 18 randomly picked strains were cultivated on the PDA plates for seven days, at room temperature and 50 g of wheat grains were used for inoculum. The grains were kept (immersed) in the distilled water (22.5 mL) in the 250-mL flasks for 24 h, until they reached 45% moisture content. Thereafter, the wheat grains were autoclaved at 121 °C for 30 min. Small fragments of agar inoculated with *F. graminearum* mycelium were added to the autoclaved wheat grains (50 g), whereas the agar fragment without mycelium was used as a negative control. After inoculation the samples were incubated at 25 °C for 3 weeks, and the obtained cultures were firstly dried for 72 h at 50 °C and thereafter ground to the fine powder used for mycotoxin analyses.

The DON content (sum of the 3-ADON, DON, 3-glucosil-DON and 15-ADON) was determined with Celer Tecna^®^ DON ELISA kit (Tecna S.r.l., Trieste, Italy) according to the manufacturer’s instructions. The DON dosage range was 40–5000 μg kg^−1^, with the detection limit for wheat of 40 μg kg^−1^. In the samples with >5000 μg kg^−1^, the extract was diluted 5 times (1:4) in 70% methanol, in order to obtain a dosage range of 20–25,000 μg kg^−1^.

## Figures and Tables

**Table 1:** The detailed data on *F. graminearum* strains.

Strain	Wheat Variety (Host)	Year of Isolation	*F. graminearum* Genotypes			
			TRI13 Primers		TRI3 Primers	
			DON	NIV	3-ADON	15-ADON
IZS 146	Pobeda	2005	+	−	−	+
IZS 147	Pobeda	2005	+	−	−	+
IZS 148	Pobeda	2005	+	−	−	+
IZS 149	Pobeda	2005	+	−	−	+
IZS 150	Pobeda	2005	+	−	−	+
IZS 151	Pobeda	2005	+	−	−	+
IZS 152	Pobeda	2005	+	−	−	+
IZS 153	Pobeda	2005	+	−	−	+
IZS 243	Pobeda	2008	+	−	−	+
IZS 244	Pobeda	2008	+	−	−	+
IZS 250	Pobeda	2008	+	−	−	+
IZS 251	Pobeda	2008	+	−	−	+
IZS 252	Pobeda	2008	+	−	−	+
IZS 254	Pobeda	2008	+	−	−	+
IZS 255	Pobeda	2008	+	−	−	+
IZS 256	Pobeda	2008	+	−	−	+
IZS 257	Pobeda	2008	+	−	−	+
IZS 258	Pobeda	2008	+	−	−	+
IZS 362	Simonida	2015	+	−	−	+
IZS 363	Simonida	2015	+	−	−	+
IZS 364	Simonida	2015	+	−	−	+
IZS 365	Simonida	2015	+	−	−	+
IZS 366	Simonida	2015	+	−	−	+
IZS 367	NS40S	2015	+	−	−	+
IZS 368	NS40S	2015	+	−	−	+
IZS 369	NS40S	2015	+	−	−	+
IZS 370	NS40S	2015	+	−	−	+
IZS 371	NS40S	2015	+	−	−	+
IZS 372	Simonida	2015	+	−	−	+
IZS 373	NS40S	2015	+	−	−	+
IZS 374	NS40S	2015	+	−	−	+
IZS 375	NS40S	2015	+	−	−	+
IZS 376	NS40S	2015	+	−	−	+
IZS 377	Simonida	2015	+	−	−	+
IZS 378	NS40S	2015	+	−	−	+
IZS 379	NS40S	2015	+	−	−	+
IZS 380	NS40S	2015	+	−	−	+

DON, deoxynivalenol. NIV, nivalenol. 3-ADON, 3-acetyldeoxynivalenol. 15-ADON, 15-acetyldeoxynivalenol. +, products amplified of 234 bp (DON) and 610 bp (15-ADON). −, no amplification.

**Table 2:** Toxigenic potential for DON production of tested *F. graminearum* strains.

*F. graminearum* Strains	Wheat Variety	Year of Isolation	DON (µg kg^−1^)
IZS 146	Pobeda	2005	>25,000
IZS 147	Pobeda	2005	>25,000
IZS 148	Pobeda	2005	>25,000
IZS 149	Pobeda	2005	23,830
IZS 150	Pobeda	2005	23,610
IZS 151	Pobeda	2005	20,020
IZS 243	Pobeda	2008	>25,000
IZS 244	Pobeda	2008	2061
IZS 250	Pobeda	2008	21,460
IZS 251	Pobeda	2008	20,260
IZS 252	Pobeda	2008	>25,000
IZS 254	Pobeda	2008	6130
IZS 362	Simonida	2015	9250
IZS 363	Simonida	2015	3089
IZS 364	Simonida	2015	>25,000
IZS 367	NS40S	2015	7500
IZS 368	NS40S	2015	7420
IZS 369	NS40S	2015	21,050
Control	-	-	<40

DON, deoxynivalenol.

**Table 3:** Sequences of primers used in PCR assays.

Gene		Primer	Sequence (5′–3′)	Size (bp)
*FG16*		FG16F/FG16R	CTCCGGATATGTTGCGTCAA GGTAGGTATCCGACATGGCAA	400–500
*TRI13*		TRI13F/TRI13R	TACGTGAAACATTGTTGGC GGTGTCCCAGGATCTGCG	234 or 415
*TRI3*	common	3CON	TGGCAAAGACTGGTTCAC	-
	specific	3NA	GTGCACAGAATATACGAGC	TRI NIV 840
		3D15A	ACTGACCCAAGCTGCCATC	15-ADON 610
		3D3A	CGCATTGGCTAACACATG	3-ADON 243
